# High-Flow Vascular Malformation of Ear: A Case Report

**Published:** 2018-05

**Authors:** Ankit Gupta, Shyam Gupta, Akhil Kumar, Sameek Bhattacharaya, Manoj Jha, Vinay Tiwari

**Affiliations:** 1 PGIMER and RML Hospital, Delhi, India; 2 Indiapgimer and RML Hospital, Delhi, India

**Keywords:** Arteriovenous, Vascular, Malformation, External ear, Embolization

## Abstract

Vascular anomalies are categorized into vascular tumours or vascular malformations on the basis of clinical features and histopathology. The literature regarding high flow arteriovenous malformations of the auricle is rare. A case of a patient clinically diagnosed with an arteriovenous malformation of the external ear and was managed with preoperative embolization, surgical excision and reconstruction of ear with split thickness skin graft, is presented. The pathogenesis, clinical presentation, diagnostic work up, radiological intervention and management options regarding arteriovenous malformations are discussed. Total cure is an illusion and rarely achieved in high flow high grade vascular malformation with nearly 98% recurrence reported in 5 years. Patient followed for next 3 months with no evidence of recurrence.

## INTRODUCTION

Vascular anomalies are categorized into vascular tumours or vascular malformations on the basis of clinical features and histopathology, as given by Mulliken and Glowacki.^1^ We are reporting a case of Schobinger grade III gradually expanding high flow vascular malformation of left ear with skin changes in a 24-year old male. Diagnosis was made on history and clinical examination and MRI with MR angiography was done to define extent of malformation and delineate the feeding arteries. We managed it with preoperative coil embolization by percutaneous arterial angiography followed by surgical excision of malformation after 48 hrs and reconstruction of ear in the single stage. Patient was followed for 3 postoperative months with only complication of graft loss which was re-grafted. There is no recurrence of vascular malformation.

## CASE REPORT

**Fig. 1:**
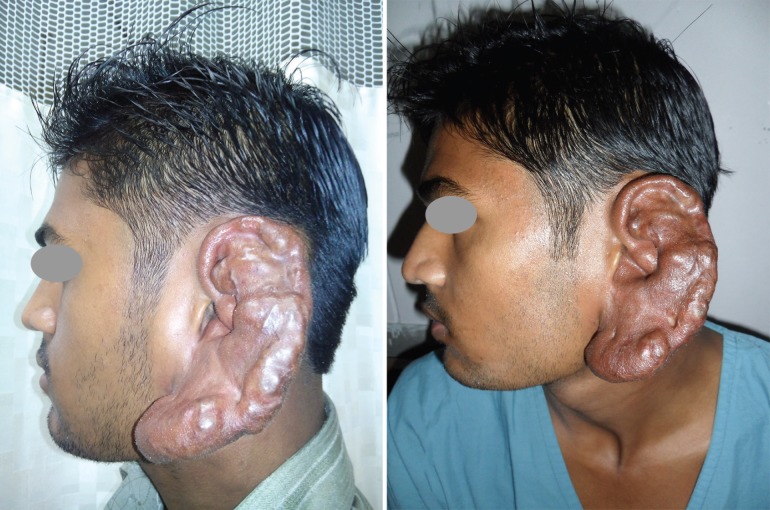
Left ear with skin discoloration. extending from the helical rim to lobule, neck inferior to the ear and post auricular region

A 24-year old male patient presented with history of gradually increasing size of left ear with skin discoloration. On examination it was a pulsatile, compressible, spongy, non-tender swelling with pigmentary changes of skin. Thrill was easily felt and continuous bruit was heard on auscultation. Patient denied bleeding, ulceration or discharge from the lesion. The malformation was involving the whole external ear extending from the helical rim to lobule, neck inferior to the ear and post auricular region. External auditory canal and tympanic membrane were normal. Clinical diagnosis of high flow arterial malformation was made on history and examination (Figure 1). 

**Fig. 2:**
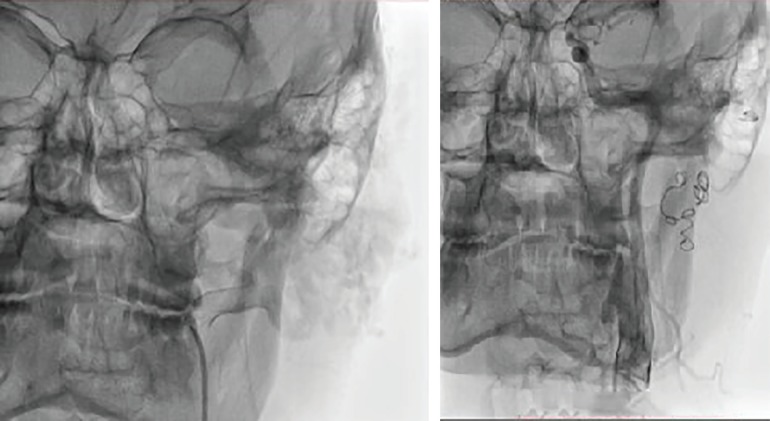
MRI and MR Angiography showing feeding vessel arising from the left external carotid artery

**Fig. 3:**
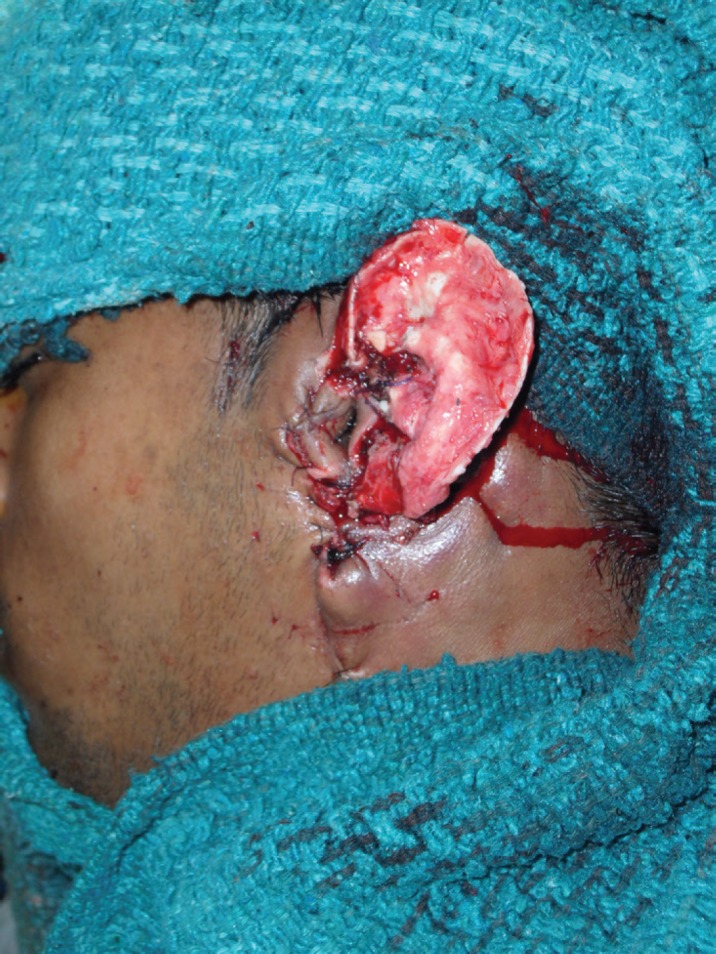
Preoperative coil embolization done to reduce vascularity and to obtain blood less field during excision.

**Fig. 4:**
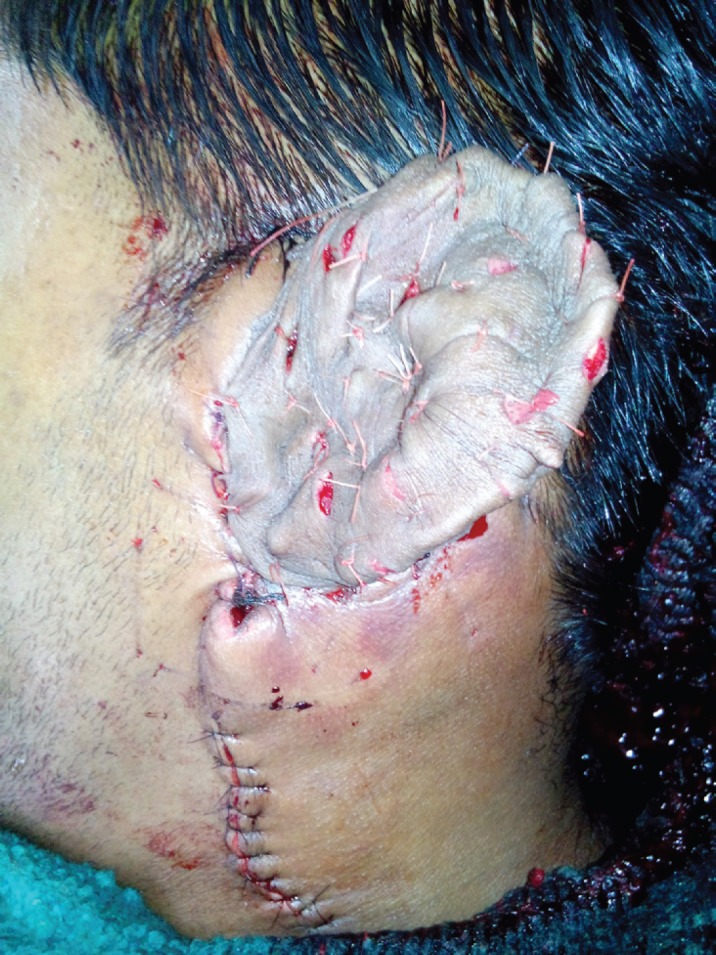
The surgery scheduled 48 hours after embolization

**Fig. 5:**
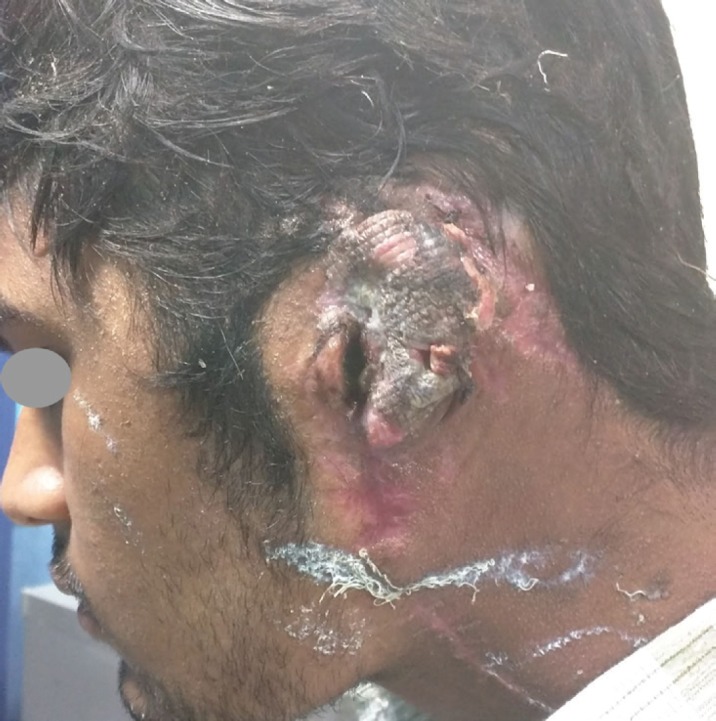
Per-operative ligation of all the large tortuous blood vessels around the affected ear and whole resection of lesion along with skin, subcutaneous tissue preserving the auricular cartilage with intact perichondrium

**Fig. 6:**
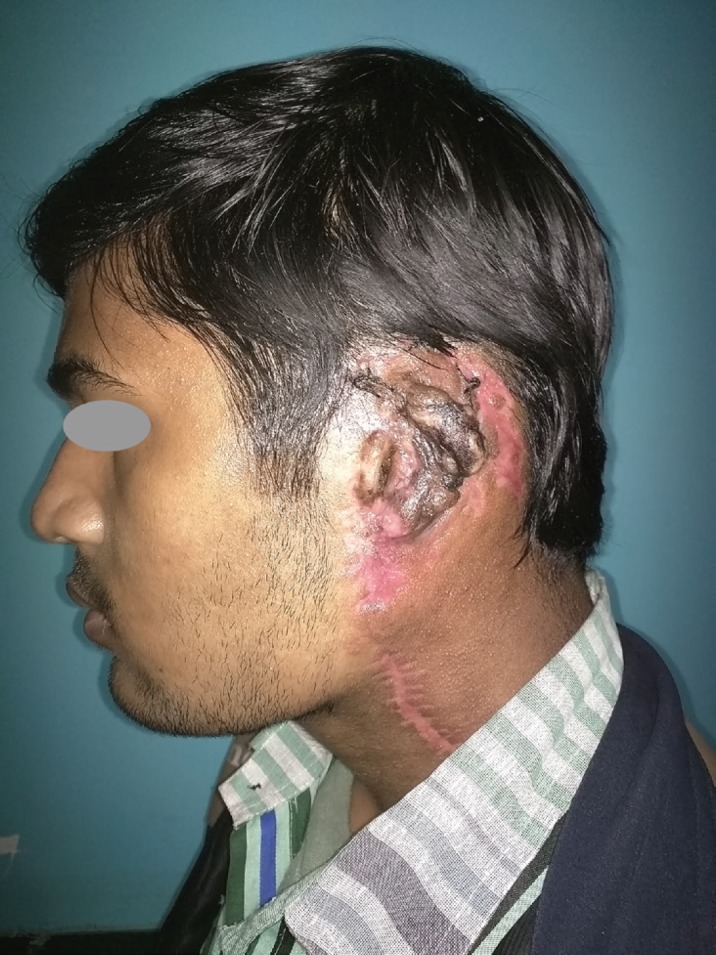
Reconstruction done with split thickness skin graft harvested from thigh covering the ear cartilage

The diagnosis was confirmed by MRI and MR Angiography which showed feeding vessel arising from the left external carotid artery (Figure 2). Preoperative coil embolization was done to reduce vascularity and obtain blood less field during excision (Figure 3). The surgery was scheduled 48 hours after embolization (Figure 4). Per-operatively all the large tortuous blood vessels around the affected ear were ligated. The whole lesion was resected along with skin, subcutaneous tissue preserving the auricular cartilage with intact perichondrium (Figure 5). Reconstruction done with split thickness skin graft harvested from thigh covering the ear cartilage (Figure 6). 

There was partial graft loss in subsequent days but the cartilage was found viable which was re-grafted. Patient was followed for three months with aesthetically acceptable ear. The surgical procedure could be carried out with precision and the auricular cartilage could be preserved with intact perichondrium because the bleeding was controlled by pre-operative embolization. In spite of this there was more than 500 ml blood loss. Post operatively patient lost a significant amount of cartilage as expected due non-vascular skin graft used as cover. This can be well appreciated in the follow up pictures. (Figure 7,8) In spite of offering the patient a reconstruction for the ear, he chose not to go for ear reconstruction as of now and is happy without the grotesque deformity.

## DISCUSSION

Vascular anomalies are classified according to the age of presentation and their natural course. The exact pathogenesis of an arteriovenous malformation (AVM) has not been clearly defined. One theory postulates that these malformations arise due to err in foetal development during 4^th^ to 6^th^ gestation week as a result of the failure of regression of arteriovenous channels in the primitive retiform plexus. It has also been postulated that local ischemia plays a role in pathogenesis. It is well known that an arteriovenous malformation enlarges rapidly following proximal ligation. It is thought that this may be the cause of enlargement of the lesions following trauma area.^2^

Proper management of vascular lesions makes it imperative for the treating surgeon to clearly comprehend the distinction between a vascular tumour and vascular malformation. Arterial malformations are comparatively rarer than venous malformations. At the same time these high flow lesions are challenging to manage due to uncontrolled bleeding, involvement of vital structures and difficulty in complete excision. An absent capillary bed causes shunting of blood directly from the arterial to-venous circulation, through a fistula (direct connection of an artery to a vein) or nidus (abnormal channels bridging the feeding artery to the draining veins). The most common site of extracranial AVM is the head and neck, followed by the limbs, trunk, and viscera.^3^

Fifty percent of these occur in the head and neck, out of which 69% are seen in the midfacial area.^2^ Therefore, most surgeons’ experience with such a wide variety of lesions would be limited, leading to therapeutic uncertainties. It can be best managed with multiple disciples involving good co-ordination among head and neck surgeons, plastic surgeons, cardiologists and interventional radiologists. Plain x-rays and CT scan are of limited value. MRI and MR angiography are best used to define the extent, confirm the diagnosis and planning for excision. Angiography is reserved when there is diagnostic uncertainty or the excision is planned. Angiographic appearance of AVM is tortuous, dilated, arteries with arteriovenous shunting and dilated draining veins.^4^

The nidus is angiographically seen as tortuous, small vessels, with occasionally ill-defied larger contiguous vascular spaces. Low-flow malformations are best managed by observation unless symptomatic which then treated with excision. Most of the vascular malformations recur in 1^st^ year of excision and 98% recurs within 5years making the cure almost impossible. Hence the goal of treatment should be very clear in terms of outcome and patient should be counselled in detail about the recurrence. The goal must be to avoid or treat complications like bleeding, pain, ulceration and for functional deformity or disfigurement and not to attain complete cure.^5^

Treatment of choice for AVM is embolization to occlude nidus and proximal venous outflow with temporary material like gel foam, PVA, embospheres followed by surgical excision and reconstruction of deformity. Permanent liquid agents capable of permeating the nidus (n-BCA, Onyx) are employed when embolization is the primary treatment and resection is not feasible.^4^ Preoperative embolization significantly devascularizes the lesion, creates a scar that aids in surgical resection, and reduces the size of lesion. Resection of AVM has a lower recurrence rate than embolization alone; it is considered for a well-localizedlesion or to correct deformity (i.e., bleeding or ulcerated areas, labial hypertrophy).^5^

Wide extirpation and reconstruction of large, diffuse AVM should be exercised with caution as the resultant deformity is worse than the original and cure is rare. Blood loss during resection can be reduced using hypotensive anaesthesia, normo-volumic hemodilution, extracorporeal cardiopulmonary bypass with deep hypothermic circulatory arrest.^6^ Surgical ligation of proximal feeding vessels should be discouraged as it not only aggravates the lesion by promoting new collaterals but also precludes future embolization in case of recurrence which is inevitable by blocking the access to nidus. Histopathological diagnosis in rarely necessary and is needed to rule out malignancy if imaging finding are equivocal. We recommend all patients of medium to large high flow AVM to undergo preoperative angioembolisation if feeding vessels can be identified followed by resection after 24 to 72 hrs.

## CONFLICT OF INTEREST

The authors declare no conflict of interest.
